# Translational impact of machine learning-driven predictive modeling with pathway-based plasma metabolomic biomarkers for lung cancer detection

**DOI:** 10.3389/fonc.2025.1718863

**Published:** 2026-01-22

**Authors:** Eyad Himdiat, Jean-François Haince, Rashid A. Bux, Guoyu Huang, Paramjit S. Tappia, Bram Ramjiawan, Maria Vaida

**Affiliations:** 1 Department of Analytics, Harrisburg University of Science and Technology, Harrisburg, PA, United States; 2 BioMark Diagnostic Solutions Inc., Quebec, QC, Canada; 3 BioMark Diagnostics Inc., Richmond, BC, Canada; 4 Asper Clinical Research Institute and Albrechtsen Research Centre, St. Boniface Hospital, Winnipeg, MB, Canada; 5 Department of Pharmacology & Therapeutics, Max Rady College of Medicine, University of Manitoba, Winnipeg, MB, Canada

**Keywords:** biomarker, early detection, glutaminolysis, lung cancer, machine learning, metabolomics, tryptophan metabolism

## Abstract

**Objectives:**

The detection of lung cancer at its early stages remains essential for better survival outcomes, but current diagnostic approaches show limited sensitivity and often suffer from poor generalizability and a lack of interpretability.

**Methods:**

This retrospective study develops a machine-learning pipeline that integrates plasma metabolite measurements with pathways to derive a pathway-informed biomarker panel for lung cancer screening.

**Results:**

Using 800 plasma samples from the Cooperative Human Tissue Network biobank (586 cancer, 214 controls) with 166 metabolites and 60 derived pathways, we identified a subset of 41 predictors (9 pathways, 26 metabolites, 6 demographic variables) through an ensemble selection framework. Several models were tested with the Support Vector Machines (SVM) model, achieving the best results. The model delivered an overall 97% accuracy with a ROC AUC of 0.97 on this subset. After eliminating pathway-related metabolites from the initial dataset, feature selection reduced the number of variables from 170 to 41, retaining biological relevance and minimizing overfitting. The glutaminolysis and tryptophan metabolism pathway analysis yielded the most enhanced biological indicators.

**Conclusions:**

This noninvasive, interpretable approach using plasma panel could facilitate cost-effective, early-stage lung cancer screening for at high-risk population cohort, with strong translational potential in clinical settings. Future work should focus on multi-center validation, prospective validation, assessing potential longitudinal biomarker stability, and integration with other omics data to further advance precision oncology, ultimately improving early detection and patient outcomes in lung cancer management.

## 1 Introduction

Lung cancer remains the leading cause of cancer-related mortality worldwide, with an estimated 234,580 new cases and 125,070 deaths projected in the United States in 2024, according to the American Cancer Society (1). The five-year survival rate highlights the urgency for early detection, with non-small cell lung cancer (NSCLC) at 28% and small cell lung cancer (SCLC) at 7%. Smoking, defined here as >20 pack-years or former smoking within the past 15 years, remains the primary risk factor, accounting for 80–90% of lung cancer deaths; nevertheless, nonsmokers still face substantial lifetime risk, estimated at roughly 1 in 16 men and 1 in 17 women, according to the CDC (2). Despite technological advances, current diagnostic tools, including chest radiographs, computed tomography (CT), positron emission tomography (PET), and tissue biopsies, are limited by invasiveness, cost, and diagnostic ambiguity, often leading to missed or delayed identification of early-stage disease (3). These shortcomings have prompted growing interest in non-invasive, blood-based diagnostic strategies, especially through metabolomics.

Metabolomics, the comprehensive profiling of small molecules from cellular metabolism, offers a powerful lens into cancer biology. It can reveal disruptions in key metabolic pathways, such as aerobic glycolysis (Warburg effect), glutaminolysis, and tryptophan metabolism, which are known to augment tumor proliferation, immune evasion, and resistance to therapy (4–6). Machine learning (ML)–driven metabolomics models have demonstrated potential in classifying lung cancer patients (7–9). Yet, most existing models rely on individual metabolites, omitting pathway-level context, which diminishes biological interpretability and limits translation utility. Recent multi-cohort studies and systematic reviews echo this limitation. Although metabolite panels perform well within specific populations, they often lack reproducibility across cohorts due to small sample size and high sample heterogeneity (10). Integrating proteomic and metabolomic data have shown promising results but lacks the interpretability of pathway-level linkage and functional pathway annotations (11–14).

To address these shortcomings, we proposed a pathway-informed diagnostic framework that transforms metabolite-level features into curated pathway-level representations using the Human Metabolome Database (HMDB) (15). This approach aimed to improve both predictive accuracy and biological clarity in identifying lung cancer. Using 800 plasma samples from the Cooperative Human Tissue Network (CHTN, USA), including 586 lung cancer patients and 214 healthy controls, we applied a two-round feature selection and modeling pipeline to investigate these challenges with a wide range of metabolomic and demographic variables. We hypothesized that incorporating pathway-level metabolomic features into a ML model will (i) improve classification accuracy compared to models using individual metabolites, (ii) identify key metabolic pathways such as glutaminolysis and tryptophan metabolism as strong predictors of lung cancer presence and progression, and (iii) enhance model interpretability and clinical relevance through biologically meaningful insights.

## 2 Materials and methods

### 2.1 Study cohort and sample collection

**Figure 1:**
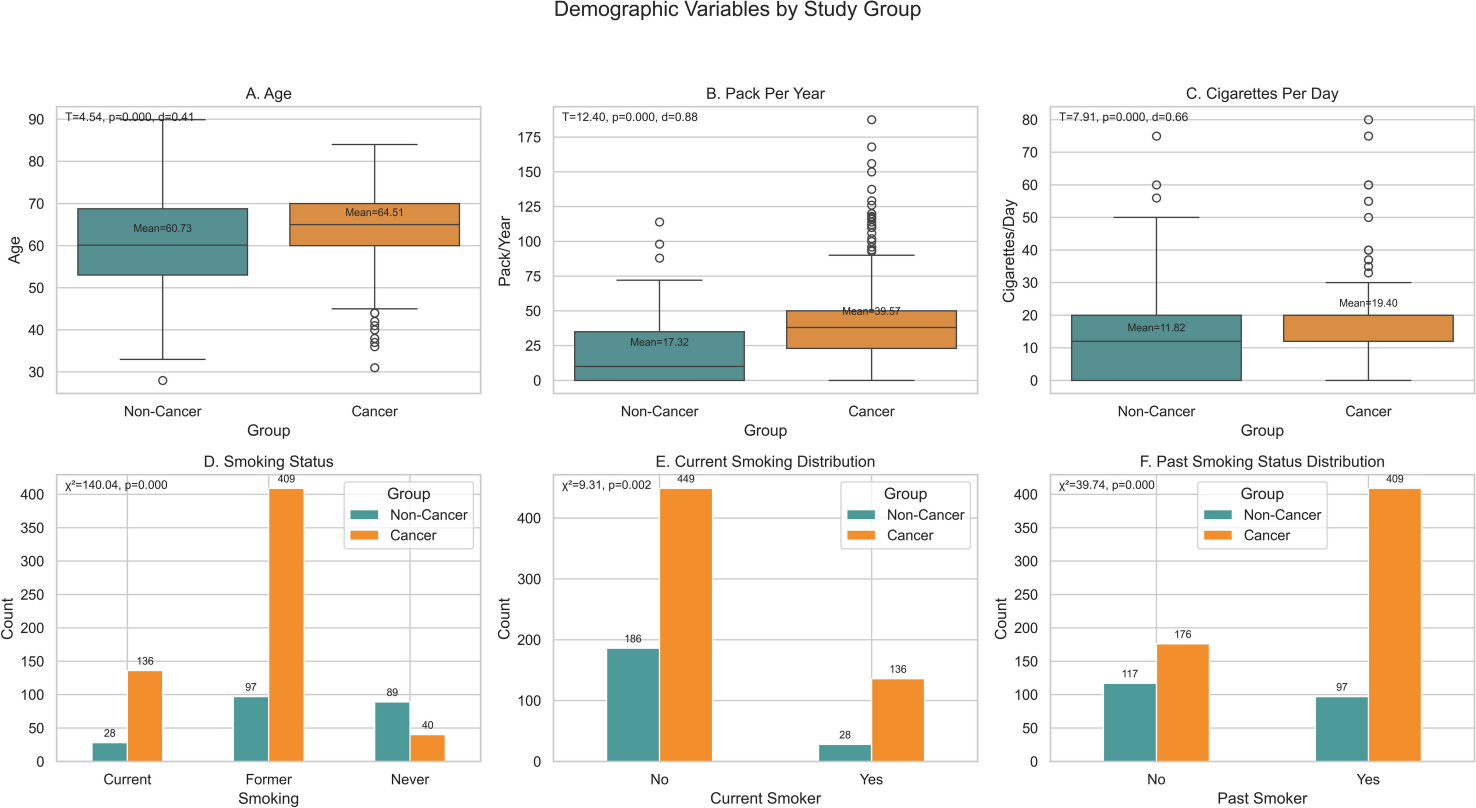
Demographic characteristics by study group. Six demographic variables identified as important features in model performance are compared between the non-cancer and cancer groups. Boxplots show **(A)** age, **(B)** Pack Per Year, and **(C)** Cigarettes Per Day. Bar charts show **(D)** Smoking Status (Current/Former Smoking/Never), **(E)** Current Smoker Distribution (Yes/No), and **(F)** Past Smoking Status Distribution (Yes/No). Statistical annotations indicate group differences.

The dataset comprised 800 plasma samples obtained from the CHTN, including 586 patients with histologically confirmed lung cancer (encompassing NSCLC and SCLC across stages I–IV, and 214 age and sex matched healthy controls with no cancer history. Plasma samples were collected under fasting conditions, stored at -80°C to preserve metabolite integrity, and analyzed using the DI-LC/MS/MS TMIC PRIME assay at The Metabolomics Innovation Centre, Canada. Calibration curves with internal standards ensured accuracy, and quality control measures, including duplicate analyses and blanks maintained a coefficient of variation below 10% for all metabolites. The assay quantified 166 unique metabolites, including amino acids, acylcarnitines, biogenic amines, glycerophospholipids, and sphingolipids, using an Agilent 1290 Infinity LC system coupled to a Sciex QTRAP 5500 mass spectrometer. Calibration curves with internal standards ensured accuracy, and quality control measures, including duplicate analyses and blanks, maintained a coefficient of variation below 10% for all metabolites, as recommended for high-throughput metabolomics (16). Demographic variables collected included age (in years), sex (male/female), smoking status (current, past, never), pack-years (calculated as packs smoked per day multiplied by years smoked), cigarettes per day, and comorbidities such as chronic obstructive pulmonary disease or diabetes, recorded from patient medical histories. Demographic variables revealed key differences between groups, with cancer cases being older and having higher smoking exposures. These findings are depicted in **Figure 1** and **Table 1**, offering a visual summary of group differences and statistical tests (t-values, p-values, Cohen’s d).

**Table 1:** Distribution of demographic and smoking variables.

Characteristic	Non-cancer controls (n = 214)	Lung cancer cases (n = 586)
Age, years, mean ± SD	60.7 ± 11.0	64.5 ± 8.6
BMI, kg/m², mean ± SD	26.4 ± 4.7	27.2 ± 5.4
Sex, n (%)		
Female	98 (45.8%)	308 (52.6%)
Male	116 (54.2%)	277 (47.4%)
Race, n (%)		
Caucasian	211 (98.6%)	510 (87.2%)
Non-Caucasian	3 (1.4%)	75 (12.8%)
Smoking status, n (%)		
Never	89 (41.6%)	40 (6.8%)
Current	28 (13.1%)	136 (23.2%)
Former	97 (45.3%)	409 (69.9%)

Ethics approval was obtained from the University of Manitoba Health Research Ethics Board (Ethics File #: H2012:334) prior to the implementation of the study. Research ethics approval was also obtained from the University of Alberta (Study ID: Pro00093715) to conduct the metabolomic studies in Edmonton. The metabolomics dataset analyzed in this study is subject to data-use restrictions due to patient privacy protection and licensing agreements with BioMark Diagnostics Inc.

### 2.2 Feature selection and model development

From the 166 metabolites, 60 pathway-level features were derived from HMDB. Each pathway feature was calculated by averaging the z-score-normalized concentrations of 2–13 metabolites associated with a specific biochemical pathway, as defined by HMDB annotations and cross-validated with KEGG and Reactome databases to ensure accuracy. Pathways included glutaminolysis and cancer (linked to energy production and redox homeostasis), tryptophan metabolism (immune regulation), congenital lactic acidosis (mitochondrial dysfunction), oncogenic action of succinate (epigenetic changes in tumorigenesis), arginine and proline metabolism (cellular stress responses), transfer of acetyl groups into mitochondria (energy metabolism), glycogenosis (glycogen storage disorders), glycogen storage disease Type VII (Tarui disease, affecting glycolysis), and Fanconi-Bickel syndrome (Type XI, impairing glucose/galactose metabolism) were included in the dataset. To avoid redundancy, individual metabolites contributing to a pathway were excluded from the dataset, resulting in a final set of 170 features, comprising 60 pathways, 104 remaining individual metabolites, and 6 demographic variables.

**Figure 2:**
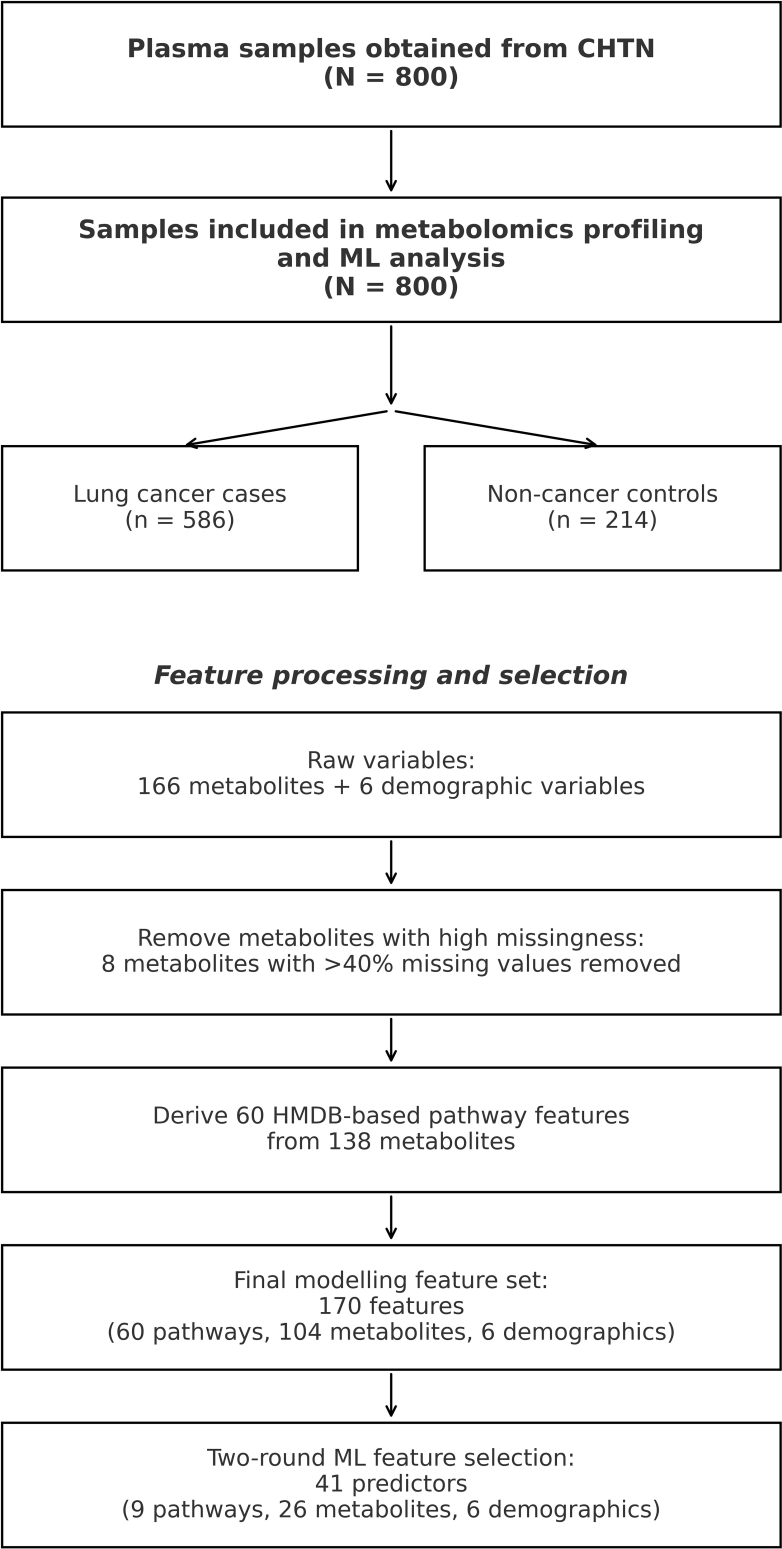
Study cohort flow and feature processing pipeline. Plasma samples were obtained from the Cooperative Human Tissue Network (CHTN) (N = 800) and included in downstream metabolomics profiling and model development (586 lung cancer cases and 214 non-cancer controls). Initial profiling measured 166 metabolites and 6 demographic variables. Eight metabolites with >40% missing values were removed prior to pathway feature engineering. A total of 60 pathway features were derived from 138 metabolites based on HMDB annotations. The final modeling feature set included 170 predictors (60 pathways, 104 metabolites, and 6 demographics), followed by two-round feature selection resulting in 41 predictors used for model training and evaluation.

Data preprocessing involved dropping features with more than 40% missing values (8 metabolites were removed), with the remaining missing metabolites being imputed using the instrument’s detection limit. Demographic variables were imputed using mean values. Continuous variables were standardized (z-scores), and outliers exceeding five standard deviations were capped to minimize noise. To address class imbalance, SMOTE was applied to the training set, generating additional synthetic control samples. The dataset was stratified into training (80%, n = 640), validation (10%, n = 80), and testing (10%, n = 80) sets, using stratified sampling across ten random seeds to maintain class proportions consistently. The modeling was implemented in Python 3.9 (scikit-learn v1.0.2, XGBoost v1.5.0). In the feature selection stage, five supervised algorithms including Logistic Regression with L2 regularization (C = 1.0), SVM (linear, C = 1.0, max_iter=1000), Decision Trees (criterion=Gini, max_depth=5), Random Forests (max_depth=5), and XGBoost (100 estimators, learning rate=0.1, max_depth=5) were trained on the full 170-feature set. Hyperparameters were optimized via grid search with 5-fold stratified cross-validation. Feature importance was quantified using model-specific measures including absolute standardized coefficients for Logistic rRgression and SVM, impurity-based Gini importance for Decision Trees and Random Forest models, and gain for XGBoost. The top 30 features per model were selected, and features appearing in at least three models were combined into a consensus set of 41 features (9 pathways, 6 demographic variables, 26 metabolites). This consensus procedure prioritizes robust, cross-model signals and reduces dependence on any single algorithm. A full data preprocessing workflow is shown in **Figure 2**.

The same five models plus K-Nearest Neighbors (k = 5, Euclidean distance) were ran on this reduced 41-feature dataset with renewed hyperparameter optimization. Model performance was evaluated using accuracy, precision, recall, F1-score, and ROC-AUC via 5-fold cross-validation, with final assessment on the test set. This two-stage pipeline reduced the feature space from 170 to 41 variables while preserving or improving performance and enhancing interpretability by focusing on a stable, biologically plausible subset of predictors. Model interpretability was enhanced using SHAP (SHapley Additive exPlanations) values calculated via the SHAP library (v0.40.0), quantifying each feature’s contribution to predictions, as described by Lundberg and Lee (10). For biological contextualization, enrichment analysis was performed using Enrichr with GO, KEGG, and DisGeNET databases. Genes associated with each pathway were extracted from HMDB; the top 10 enriched terms per database were selected. Enrichment percentages were computed as the ratio of overlapping genes to the pathway’s gene set size, multiplied by 100.

## 3 Results

### 3.1 Model performance on full feature set

The ML pipeline yielded robust predictive performance, with SVM demonstrating the best results across both the full and reduced feature sets. When applied to the entire dataset, SVM achieved 96% accuracy on the test set (n=160), correctly classifying 155 instances, including 40 of 44 non-cancer cases and 115 of 116 cancer cases. Precision was 0.93 for non-cancer and 0.98 for cancer, with recall values of 0.91 and 0.99, respectively, indicating high sensitivity for detecting cancer cases, critical for clinical applications. Logistic regression followed with 94% accuracy, correctly classifying 152 instances (39/44 non-cancer, 113/116 cancer), with precision of 0.91/0.96 and recall of 0.89/0.97 for non-cancer/cancer. XGBoost attained 90.62% accuracy (precision: 0.93/0.94, recall: 0.94/0.90), outperforming random forest (88.75%, precision: 0.86/0.96, recall: 0.70/0.96) and decision trees (81.87%, precision: 0.78/0.90, recall: 0.65/0.93), which showed signs of overfitting, as evidenced by lower recall for non-cancer cases.

### 3.2 Model performance on reduced feature set

**Figure 3:**
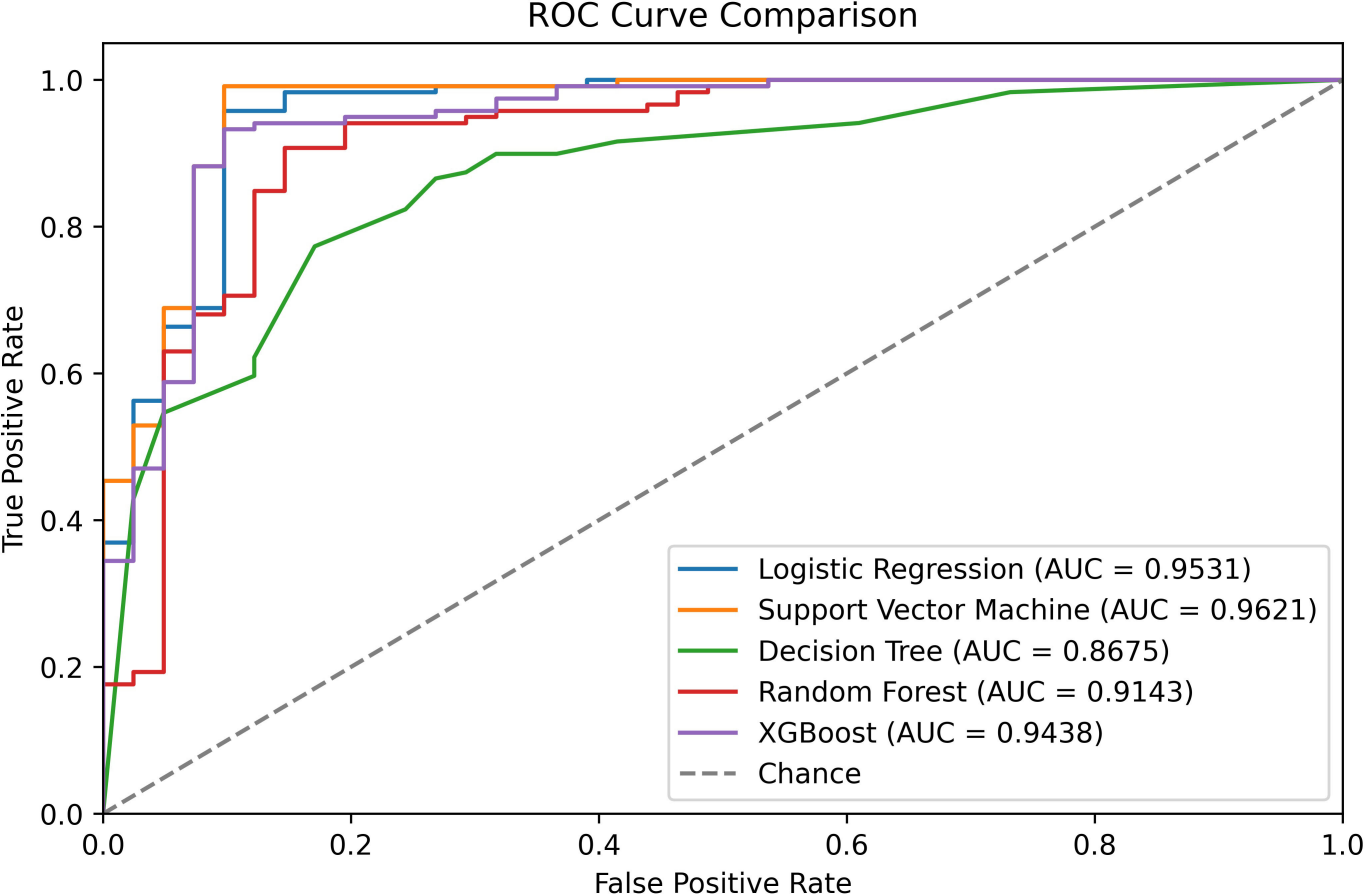
Receiver operating characteristic (ROC) curve comparison of five ML classifiers applied to pathway-annotated metabolomics data for lung cancer classification.

**Figure 4:**
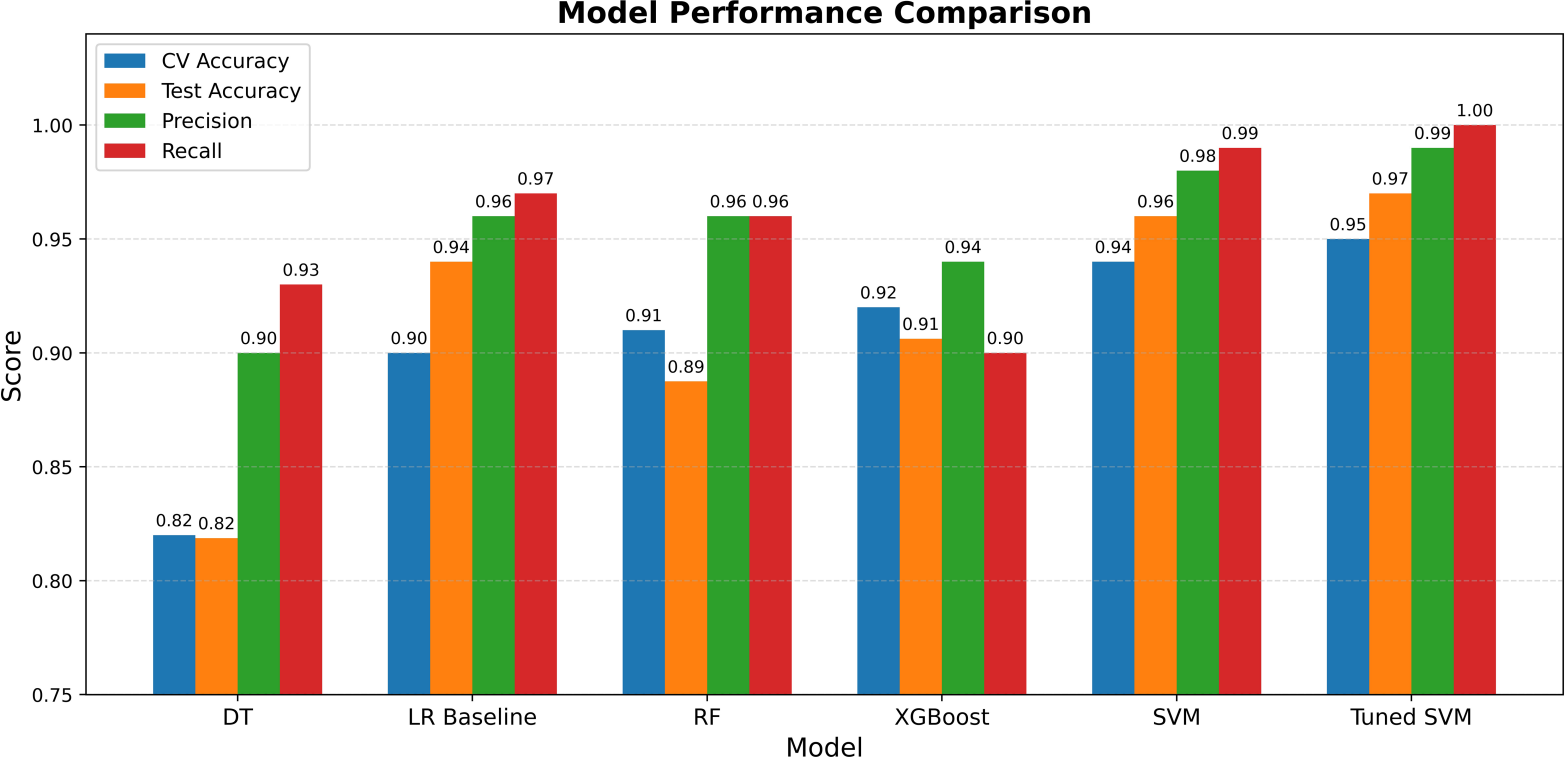
Performance comparison of six ML models. DT, Decision Tree; LR, Logistic Regression; RF, Random Forest; XGBoost, SVM, Support Vector Machine, and Tuned SVM—based on cross-validation accuracy, test accuracy, precision, and recall. The Tuned SVM achieved the highest recall, indicating its strength in identifying positive lung cancer cases key processes in cancer progression.

Using the reduced 41-feature set, SVM improved to 97% accuracy, correctly classifying 156 instances, with a ROC-AUC of 0.97, reflecting a good discriminatory ability. XGBoost achieved 95% accuracy, while KNN matched random forest at 89% accuracy, indicating inferior performance to SVM and XGBoost. The performance metrics for SVM showed an AUC of 0.97 (**Figure 3**), a macro-average F1-score of 0.95 and a weighted-average F1-score of 0.96, confirming balanced performance across classes as shown in **Figure 4**. SVM also stands out with the highest recall, indicating its strength in correctly identifying positive lung cancer cases, a critical attribute in diagnostic applications. While most models show consistent accuracy across validation and test data, the disparity in recall (especially in simpler models) highlights differences in sensitivity and potential overfitting. Models built solely on pathway features performed only at 73% accuracy, underscoring the necessity of integrating multiple feature types for optimal performance.

### 3.3 Feature importance and SHAP analysis

**Figure 5:**
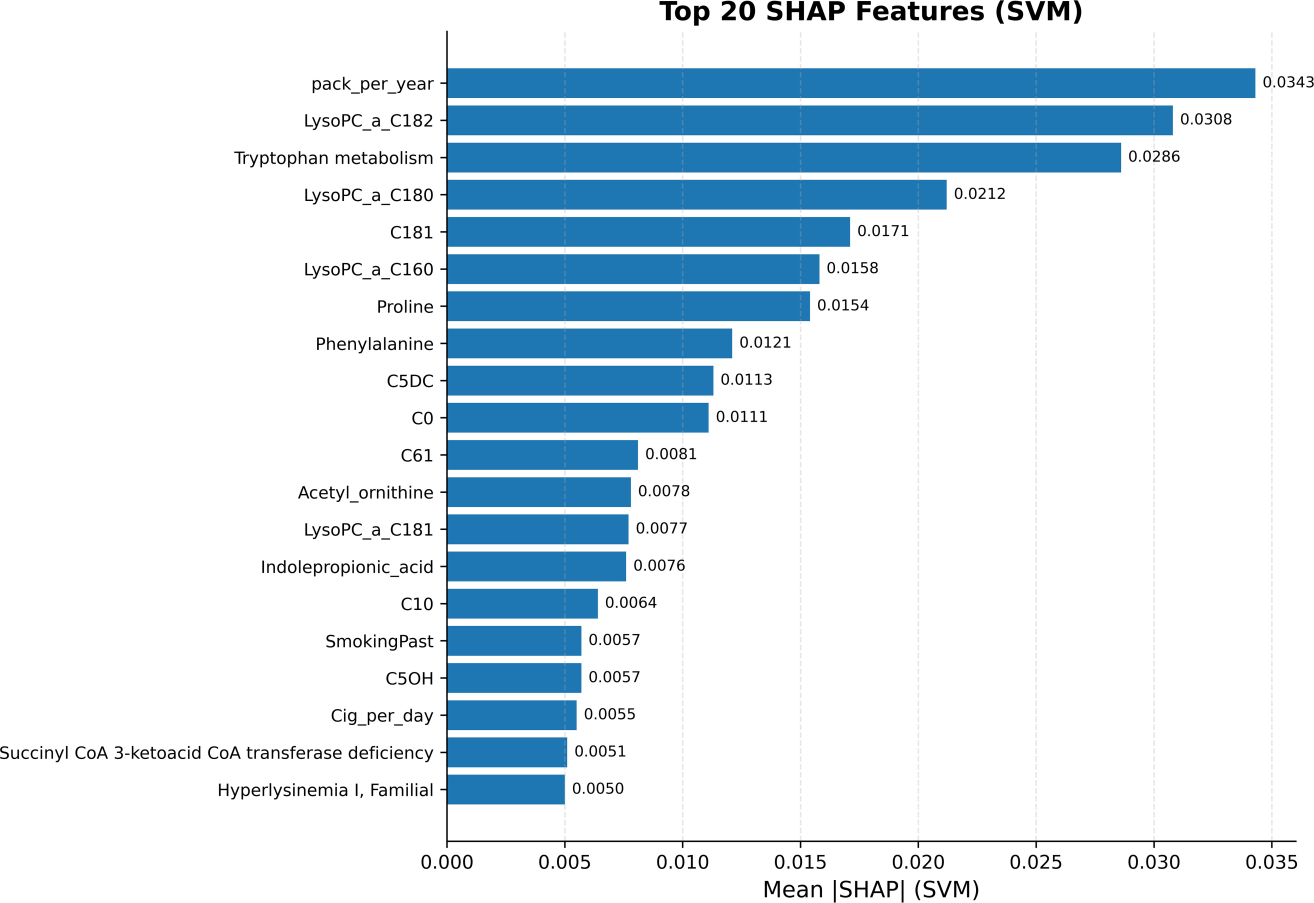
Top 20 features by mean |SHAP| for the support vector machine (SVM). Bars show the mean absolute SHAP value (importance) for the highest-ranking predictors in the 41-feature panel. Smoking exposure (pack_per_year) is the dominant contributor, followed by lipids (LysoPCs), Tryptophan metabolism pathway, amino acids (e.g., Proline, Phenylalanine), and acylcarnitines (e.g., C0, C5DC), indicating a multimodal signature spanning lifestyle factors, lipids, amino-acid metabolism, and pathway-level processes. Numeric values at bar ends represent mean absolute SHAP values.

SHAP analysis revealed that the most important factors in distinguishing between lung cancer and control included demographics, lipids, acylcarnitine, and amino acid levels, along with pathway characteristics. Cigarettes packs smoked per year emerged as the most significant factor according to SHAP values for SVM (0.0343). The most significant metabolites and pathways included LysoPCaC182 (0.0308), LysoPCaC180 (0.0212), LysoPCaC160 (0.0158), Tryptophan metabolism (0.0286), Proline (0.0154), Phenylalanine (0.0121) and acylcarnitines including C0 and C5DC. The first twenty features of SVM explained 58.1% of total importance while the top ten features explained 43.8% and the top five features explained 29.2% of total importance (**Figure 5**). The results emphasize cancer-related changes in membrane lipids and amino acid metabolism and tryptophan/kynurenine signaling pathways while pathway features enhance the understanding of results beyond single metabolite analysis. Models trained with pathway features alone achieved 73% accuracy which proved that the best results require combining pathway data with demographics and metabolite measurements.

### 3.4 Pathway enrichment analysis

Enrichment analysis via Enrichr validated the biological relevance of the nine pathways. GO Biological Process terms displayed in **Table 2** showed high enrichment for pyruvate metabolism (88.89%) and glycolysis (70.37%), aligning with the Warburg effect, a hallmark of cancer metabolism characterized by enhanced glycolytic activity (17). KEGG pathways (**Table 3**) highlighted glycolysis/gluconeogenesis (88.89%), citrate cycle (66.33%), and HIF-1 signaling (66.67%), reflecting the hypoxic tumor microenvironment prevalent in lung cancer (18). The clinical significance of DisGeNET enrichment analysis (**Table 4**) revealed important disease-related associations with neurological and metabolic disorders. The most significant disease terms included Seizures (45.83%), Mitochondrial Diseases (43.33%) and Epilepsy (43.75%), indicating that cancer metabolism might share molecular pathways with specific inherited or systemic disorders (19).

**Table 2:** Enrichment percentages for the nine discovered pathways in the lung cancer dataset across gene ontology (GO) terms.

GO term	P-value	Enrichment (%)
Pyruvate Metabolic Process (GO:0006090)	1.32 × 10^-62^	88.89
Glycolytic Process (GO:0006096)	1.03 × 10^-51^	70.37
Carbohydrate Catabolic Process (GO:0016052)	1.25 × 10^-^	70.37
Glucose Metabolic Process (GO:0006006)	6.88 × 10^-22^	40.74
Dicarboxylic Acid Metabolic Process (GO:0043648)	9.30 × 10^-22^	25.00
Phosphate-Containing Compound Metabolic Process (GO:0006796)	3.94 × 10^-1^	44.44
Tricarboxylic Acid Metabolic Process (GO:00072350)	9.30 × 10^-1^	22.22
Fructose 6-Phosphate Metabolic Process (GO:0006002)	2.79 × 10^-16^	22.22
Arginine Metabolic Process (GO:0006525)	6.97 × 10^-16^	22.22
Gluconeogenesis (GO:0006094)	1.15 × 10^-1^	25.93

**Table 3:** Enrichment percentages for the nine discovered pathways in the lung cancer dataset based on KEGG pathways.

KEGG pathway	P-value	Enrichment (%)
Glycolysis/Gluconeogenesis	1.05 × 10^-^	88.89
Citrate cycle (TCA cycle)	6.90 × 10^-5°^	66.33
HIF-1 signaling pathway	1.84 × 10^-9^	66.67
Arginine biosynthesis	8.12 × 10^-33^	29.17
Arginine and proline metabolism	3.38 × 10^-31^	33.33
Central carbon metabolism in cancer	2.56 × 10^-2^	40.54
Fructose and mannose metabolism	2.72 × 10^-22^	37.04
Tryptophan metabolism	6.67 × 10^-22^	35.71
Pyruvate metabolism	4.39 × 10^-2^	33.33
Glucagon signaling pathway	9.76 × 10^-1^	37.04

**Table 4:** Enrichment percentages for the nine discovered pathways in the lung cancer dataset according to DisGeNET disease associations.

Disease term	P-value	Enrichment %
Hyperammonemia	1.85 × 10^-35^	29.17
Lethargy	2.44 × 10^-29^	25.00
Mitochondrial Diseases	3.51 × 10^-2^	43.33
Seizures	4.23 × 10^-15^	45.83
Irritation - emotion	7.21 × 10^-15^	20.83
Epilepsy	5.95 × 10^-1^	43.75
Ketotic hypoglycemia	1.09 × 10^-13^	25.93
Comatose	1.33 × 10^-12^	18.75
Encephalopathies	2.38 × 10^-13^	44.00
Urea Cycle Disorders, Inborn	3.80 × 10^-12^	18.52

**Figure 6:**
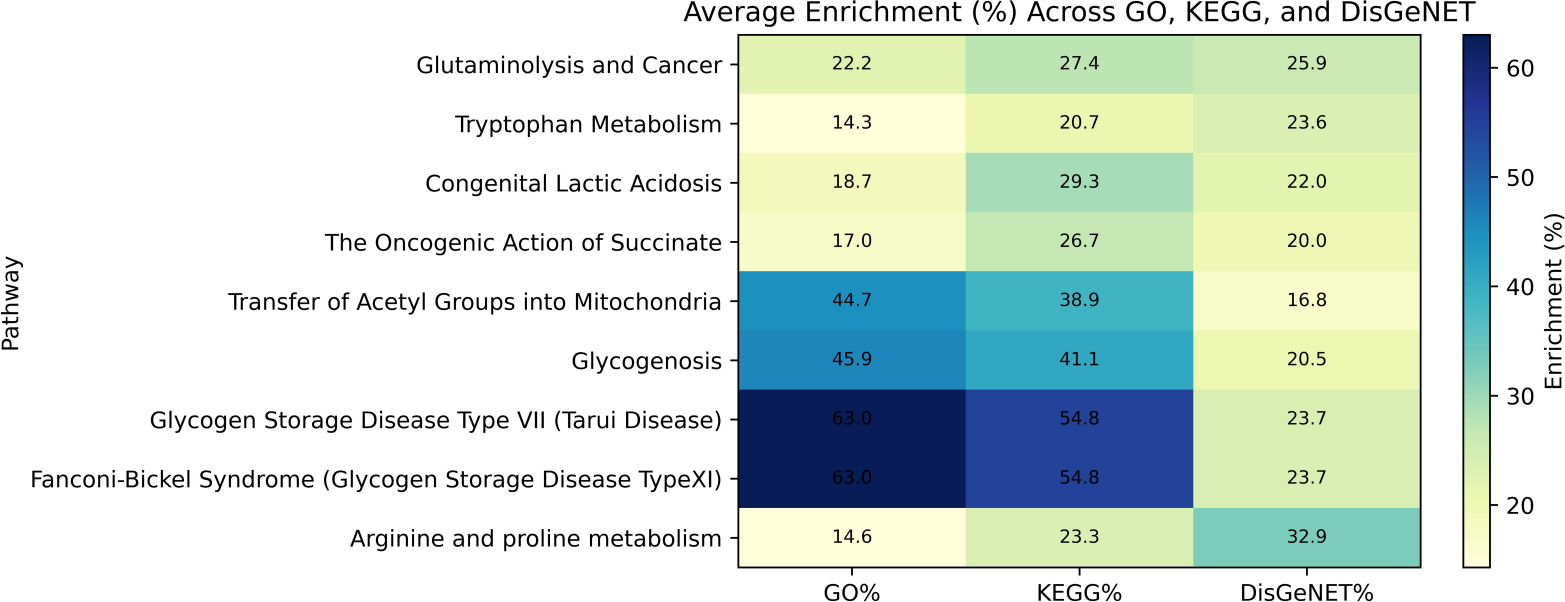
Average pathway enrichment (%) across GO, KEGG, and DisGeNET knowledgebases. Each row represents a biologically or disease-relevant pathway, and each column reflects the average enrichment score (%) within one of the three annotation sources. Strong concordance was observed for glycogen-related disorders across GO and KEGG, while DisGeNET showed relatively higher enrichment for disease-centric pathways such as Arginine and Proline Metabolism. Color intensity corresponds to enrichment percentage, with darker shades indicating higher values.

To further synthesize the enrichment results and reduce redundancy, we computed a summary-level enrichment profile for each discovered pathway by averaging the enrichment scores of the top 5 most significant terms from three biological knowledgebases: GO Biological Process, KEGG Human Pathways, and DisGeNET Disease Associations. To obtain a compact summary of the enrichment patterns across pathways, we developed a function that queries the Enrichr API for each pathway-specific gene list. For each of the three annotation libraries the API returns an ordered list of enriched terms ranked by statistical significance. We extracted the top 5 terms for each library and computed the average enrichment percentage across these terms. These average values capture a pathway’s general representation across its most statistically significant terms, smoothing the variability caused by any single annotation (**Figure 6**). Glycogen Storage Disease Type VII (Tarui Disease) and Fanconi-Bickel Syndrome (Glycogen Storage Disease Type XI) had the highest GO-based enrichment scores, which indicates that cancer might be connected to kidney glycogen accumulation (20). The highest DisGeNET enrichment score was observed in arginine and proline metabolism, which suggests its potential role in lung cancer disease phenotypes (21, 22). The pathways Glycogenosis and Transfer of Acetyl Groups into Mitochondria received equal contributions from all three sources, which indicates their importance in both biological functions and disease processes (23).

## 4 Discussion

The present study represents a new method for plasma metabolomics analysis, which combines pathway-based features from HMDB with individual metabolite data and demographic information and uses SHAP for model interpretation and GO/KEGG/DisGeNET for multi-database enrichment. The combination of these design elements produces better predictive results and improved biological and clinical understanding of metabolomics classifiers, which previously have focused on single metabolites. Compared to prior studies, this model offers robust accuracy and AUC, stemming from pathway integration, which captures complex biochemical interactions that are missed by metabolite-focused approaches. The combination of pathway-level features from HMDB with individual metabolites and demographic information leads to improved lung cancer detection. SVM achieved 97% accuracy and 0.97 ROC-AUC values superior to previous metabolomic-only models such as Guan with 0.81 (7) AUC and Shang et al. with 0.92 AUC (9). The feature selection approach decreased the number of features from 170 to 41 while maintaining biological significance and preventing overfitting which occurs when using single metabolites in studies.

The nine pathways included glutaminolysis and cancer, tryptophan, arginine and proline metabolism, congenital lactic acidosis and oncogenic succinate action, acetyl group transfer to mitochondria, glycogenosis, Tarui disease, and Fanconi-Bickel syndrome. Glutaminolysis enables tumor growth through its ability to supply biosynthetic materials and control redox reactions according to previous studies about lung adenocarcinoma and additional cancer types (24). The immune response regulation through tryptophan metabolism enables tumor evasion through IDO1 and TDO enzymes which makes this pathway a potential therapeutic focus (25). The oncogenic action of succinate leads to epigenetic transformations and metastasis development in cancers (26). The metabolic pathways of arginine and proline support cellular stress responses and extracellular matrix remodeling which are essential for tumor microenvironments (21, 22). The process of mitochondrial acetyl group transfer controls energy production and glycogen-related pathways show signs of glucose imbalance which matches cancer’s metabolic transformation patterns (23). The results from enrichment analysis supported these findings by showing connections between pathways and glycolysis and HIF-1 signaling and metabolic disorders which match current lung cancer understanding (25).

Our findings confirm metabolic hallmarks consistently reported in NSCLC and SCLC, including enhanced aerobic glycolysis (Warburg effect), glutamine dependence, and altered phosphatidylcholine metabolism, supporting the biological relevance of the identified metabolites (27–29). Our classifier also exceeds the performance of recent imaging–based machine-learning models, which report AUC values of 0.85–0.93 (30, 31), indicating stronger diagnostic accuracy. In addition, the performance of our plasma-based signature is in line with recent metabolomics studies that demonstrated early-stage detection with AUCs ranging from 0.62 to 0.96 (9, 32, 33). These consistencies support the potential value of metabolic biomarkers in complementing imaging-based methods by lowering false-positive rates in high-risk screening settings.

Compared with circulating tumor DNA (ctDNA) assays that show reduced sensitivity in stage I disease due to low analyte abundance, metabolomic profiling captures earlier systemic metabolic alterations. Large methylation-based assays and machine-learning ctDNA classifiers have reported sensitivities ranging from ~20–90% in stage I–II disease (34). Although newer lung-cancer–focused methylation panels have reported improved AUC values ranging between 0.85 and 0.95 (35), ctDNA testing remains technically demanding and is constrained by low analyte abundance in patients with small tumor burden (36). In contrast, our plasma metabolomics classifier achieved an AUC of 0.97 with 99% sensitivity and 91% specificity in this cohort, indicating strong potential for early detection in a cohort where stage I–II disease represented 88% of cases, while the remaining 12% include stages III and IV. Because metabolite changes capture both tumor metabolism and host systemic responses, metabolomics may complement ctDNA and LDCT within future multimodal screening strategies to enhance detection while reducing false-positive findings.

There are limitations to the present study. In this regard, the findings need to be validated across multiple centers involving different population cohorts and extended follow-up studies are required to verify biomarker stability throughout time. The retrospective design using samples from a single biobank may constrain generalizability to broader populations. To address this limitation, ongoing efforts include expanding the study into multi-center validation across international sites including France, seven clinical centers in Quebec, and Germany with both prospective and longitudinal follow-up to evaluate biomarker stability and real-world robustness. These future data will enable assessment of population diversity, technical variability, and clinical performance. Another limitation is that although pathway enrichment analysis supports biological plausibility, our findings remain correlative and have not been fully validated in clinical settings. Individual metabolites often participate in multiple biochemical processes and may originate from tissues beyond the tumor, making it difficult to link changes to a single pathway. Limited coverage of low-abundance intermediates and uncertainty in metabolite annotation can also affect pathway assignment (37). Future work will explore experimental validation of markers such as tryptophan, glutaminolysis, and lipid dysregulation signatures through targeted LC-MS/MS quantification, enzyme activity assays, and cancer cell functional studies to determine mechanistic contributions to tumor biology.

## 5 Conclusion

This study demonstrates that integrating HMDB-derived pathway-level features with individual metabolites and demographic variables significantly enhances the accuracy and interpretability of lung cancer detection. The SVM model’s 97% accuracy and 0.97 ROC-AUC, achieved through a two-round ML pipeline, could potentially offer a non-invasive, biologically informed tool for early screening. Pathways such as glutaminolysis and tryptophan metabolism, validated through enrichment analysis, capture critical cancer hallmarks, while smoking-related variables underscore established risk factors. The high sensitivity and specificity of this approach make it suitable for clinical settings, particularly for populations at high-risk for lung cancer. This interpretable, noninvasive plasma-based panel offers a promising tool for early-stage lung cancer screening, particularly in high-risk populations. By improving diagnostic accuracy and enabling stratification, the panel may facilitate clinical triage and personalized follow-up, potentially reducing unnecessary imaging and procedures. Integration into clinical decision pathways could expedite early detection, guide risk-based screening, and enable timely treatment interventions Future work should focus on multi-center validation, longitudinal biomarker stability, and integration with other omics data to further advance precision oncology, ultimately improving early detection and patient outcomes in lung cancer management.

## Data Availability

The metabolomics dataset analyzed in this study is subject to restrictions due to patient privacy protections and licensing/data-use agreements with BioMark Diagnostics Inc.
